# Bioaccumulation of methylmercury within the marine food web of the outer Bay of Fundy, Gulf of Maine

**DOI:** 10.1371/journal.pone.0197220

**Published:** 2018-07-16

**Authors:** Gareth Harding, John Dalziel, Peter Vass

**Affiliations:** 1 Bedford Institute of Oceanography, Department of Fisheries and Oceans, Dartmouth, Nova Scotia, Canada; 2 Environment Canada, Dartmouth, Nova Scotia, Canada; Chinese Academy of Sciences, CHINA

## Abstract

Mercury and methylmercury were measured in seawater and biota collected from the outer Bay of Fundy to better document mercury bioaccumulation in a temperate marine food web. The size of an organism, together with δ^13^ C and δ^15^ N isotopes, were measured to interpret mercury levels in biota ranging in size from microplankton (25μm) to swordfish, dolphins and whales. Levels of mercury in seawater were no different with depth and not elevated relative to upstream sources. The δ^13^ C values of primary producers were found to be inadequate to specify the original energy source of various faunas, however, there was no reason to separate the food web into benthic, demersal and pelagic food chains because phytoplankton has been documented to almost exclusively fuel the ecosystem. The apparent abrupt increase in mercury content from “seawater” to phytoplankton, on a wet weight basis, can be explained from an environmental volume basis by the exponential increase in surface area of smaller particles included in “seawater” determinations. This physical sorption process may be important up to the macroplankton size category dominated by copepods according to the calculated biomagnification factors (BMF). The rapid increase in methylmercury concentration, relative to the total mercury, between the predominantly phytoplankton (<125μm) and the zooplankton categories is likely augmented by gut microbe methylation. Further up the food chain, trophic transfer of methylmercury dominates resulting in biomagnification factors greater than 10 in swordfish, Atlantic bluefin tuna, harbour porpoise, Atlantic white-sided dolphin and common thresher shark. The biomagnification power of the northern Gulf of Maine ecosystem is remarkably similar to that measured in tropical, subtropical, other temperate and arctic oceanic ecozones.

## Introduction

Mercury is atmospherically borne, primarily in the stable gaseous form (Hg^0^), to higher latitudes by long-range aerial transport from the populated industrial areas in the northern hemisphere [1–3], where it is either oxidized to divalent compounds or combined as particulates that settle on oceanic or terrestrial surfaces [4–7]. Inorganic mercury is known to methylate in anoxic marine sediments by sulfur or iron reducing microbes and leach back into the overlying water column. Methylmercury (MeHg) is understood to be adsorbed and absorbed by aquatic organisms, chiefly microbes and phytoplankton, given their large surface area available. A low level of MeHg production occurs within the oxygen deficient depths of the open ocean [8–10] in the absence of sulfur- or iron-reducing bacteria [11]. Bacteria, viruses and phytoplankton are believed to be the primary entryway for mercury via the microbial loop of the pelagic food chain. It has been shown experimentally that mercury can be actively taken up by bacteria and methylated, with some of this MeHg released back to the seawater [12]. Phytoplankton can also accumulate MeHg actively within the cell [13,14] and this cytoplasmic MeHg is more readily transferred up the trophic chain than inorganic mercury [15,16]. Once within the lower trophic level, it is presently thought that the methylated form is transferred from prey to predator, a process known as biomagnification, reaching highest concentrations in terminal predators. This natural trophic phenomenon should be accentuated by the three- to twenty-fold increase in the atmospheric mercury load since the industrial revolution in the mid 1800s [1,17]. Recent North American regulations have reduced mercury emissions since the mid 1990s [18]. The primary concern with mercury is the bioaccumulation of MeHg in the marine food chain and its potential neurotoxicity to humans that consume seafood [19,20].

The first attempt to describe mercury biomagnification in a reasonably complete marine food web was done in the arctic in the 1990s with the aid of stable isotopes for trophic interpretations [21]. There are now several relatively complete marine food-chain studies of mercury bioaccumulation in the arctic [21–23], but fewer in the subarctic [24], temperate [25,26], subtropical [27] and tropical latitudes [28]. The present study is a descriptive field study in which we cover the transition from phytoplankton to the second and third trophic levels in greater detail than previous studies with the hope of gaining more insight into the transition from predominantly sorption to trophic transfer of mercury. Trophic status was determined by both the stable nitrogen isotope method [29] and extrapolation from organism mass based on size spectrum theory [30], together with known feeding habits. Most aquatic organisms are known to feed on prey two to three log_2_ intervals smaller than themselves [31,32]. Planktonic to nektonic organisms were sorted with a 25, 66, 125, 250……1600μm series of sieves to facilitate size spectrum trophic calculations [30]. A broad range of larger organisms were collected also by trawling, hand-lining, longlining and accidental net drowning and stranding of marine mammals to complete the upper trophic levels.

## Methods

### Study site

**Fig 1:**
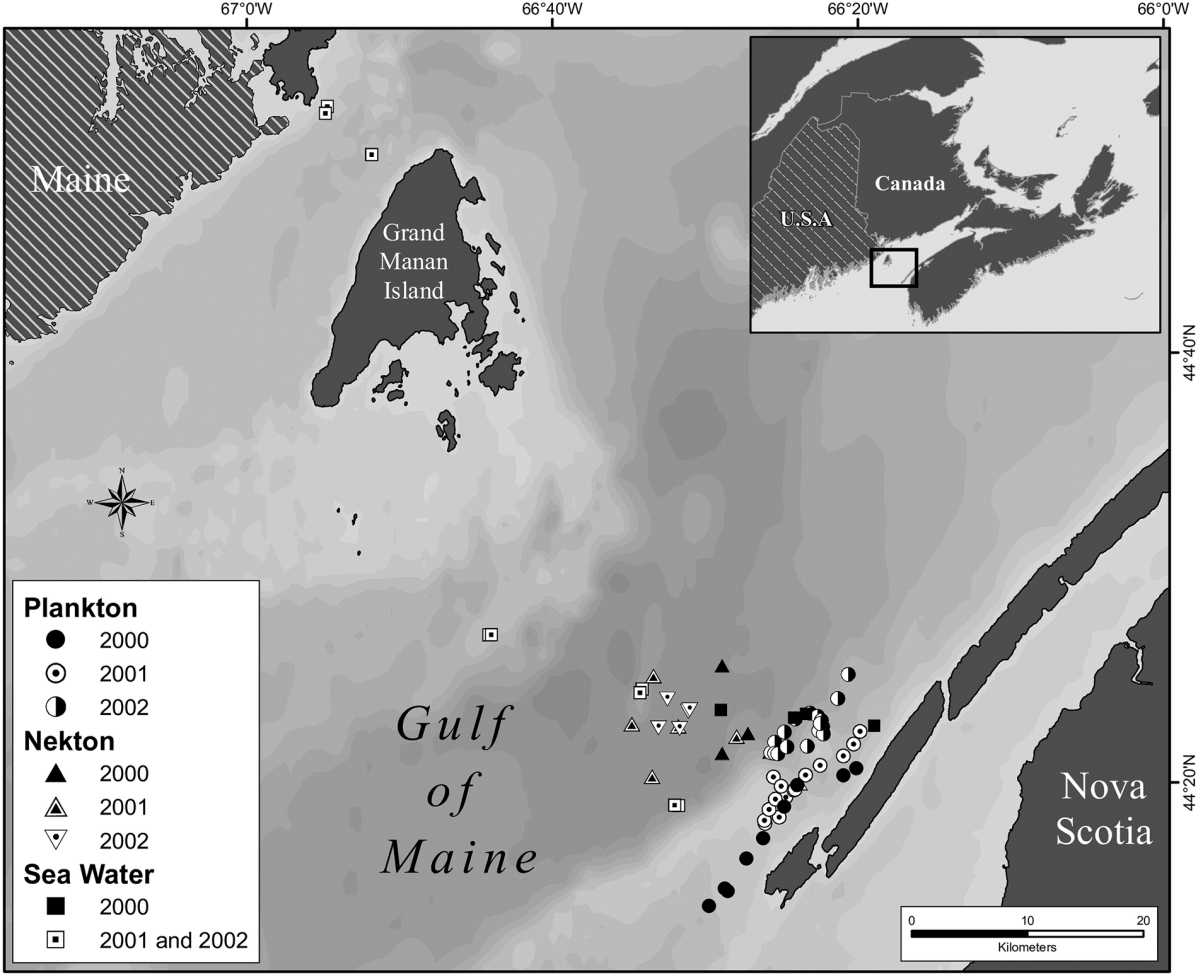
Locations in the outer Bay of Fundy where seawater, plankton and nekton collections were made, on consecutive years between 2000 and 2002, from the research vessel CCMV Navicula.

The approaches to the Bay of Fundy were chosen for our study area (Fig 1) as a relatively pristine [33–35], productive temperate embayment [36,37] that supports a diverse fishery and large predators [38–40]. The bay is a tidally well-mixed region in the Gulf of Maine [36], 45^o^N and 66^o^W, which aligns in a northeasterly direction with maximum length of ~220km and an average width of 56km and a surface area of 1.38X10^4^ km^2^. The bottom gradually deepens towards the mouth of the bay to ~200m depth where most of the biological sampling took place.

### Sample collection

Marine samples were collected on three separate years between August 21 and 24, 2000, June 12 and 21, 2001, and August 26 to September 5, 2002, at the mouth and approaches to the Bay of Fundy (Fig 1, S1, S2 and S3 Tables). In the first two years, triplicate plankton tows were taken for mercury analysis each with a 1/2m-20μm (net mouth diameter-mesh size), 3/4m-120μm and a 1m-450μm Nytex ^®^ net, equipped with TSK^®^ flow meters. This was expanded in 2002 to collect and analyse 5 replicate plankton samples from the same region. Plankton nets and their cod ends were washed before each cruise with detergent and thoroughly rinsed with fresh water before storage in plastic bags. Nets were washed down between deployments with buckets of seawater collected from the windward side of the vessel. The 20μm net was towed slowly from the side of the vessel in the upper 5m by putting the boat in and out of gear for 10 to 20 minutes, depending on the concentration of plankton present. Several grams of material were needed for mercury analysis, isotope analysis and plankton identification. Sometimes this required consecutive tows to be taken and amalgamated to obtain a sufficient sample weight. The 120μm net was towed horizontally from the side of the vessel to sample the upper 10m for 10 to 20 minutes. The 450μm net was towed obliquely throughout the upper mixed layer for 30 to 40 minutes at 1 to 2m/s. Plankton net contents were further size fractionated by passing the net contents through a waterproofed geological vibrating sieve (20cm diameter, stainless steel) assembly (Haver and Broecker, Fabr. Nr.3596^®^). This sieving apparatus was modified with upward-directed, seawater jets to clear each screen from beneath when clogging occurred [41]. The advantages of the sieving apparatus are twofold. First, the interstitial water is effectively removed which is important when mercury levels are reported on a wet weight basis for food chain studies. Secondly, a Wentworth (log _2_), stainless-steel sieve series was used for later food chain interpretations because aquatic organisms, in general, tend to consume prey two to three log _2_ intervals smaller than themselves [30]. Thus the 25μm and 63μm plankton fractions were derived from the 1/2m-20μm net contents, the 125μm and 250μm fractions from the 3/4m-120μm net and the 500 μm and 1mm fractions from the 450μm net.

Four trawls were taken with a Vass-Tucker trawl (effective mouth opening of 1m by 1.5m with a 1.5mm mesh; see [42]) for the collection of macroplankton and ichthyoplankton /nekton in 2000 and 2001. Five trawls were collected and analyzed in 2002. Clogging was not an issue with sorting the trawl catch so fractionation was achieved by pouring the contents of the cod end through stationary stacked 1mm, 2mm, 4mm, 8mm and 16mm stainless steel, geological sieves (35cm dia.). A Teflon^®^ squirt bottle filled with seawater was sometimes used to concentrate plankton on the screens for efficient collection. Plankton and ichthyoplankton samples were quickly removed by hand from the sieves, using chalk-free plastic gloves, with a Teflon spatula to minimize mercury contamination and transferred to pre-weighed Bitran ^®^ 3mil polyethylene zip bags. Plankton samples were placed in a chest freezer for storage at sea at -20°C.

Seawater was collected for mercury analysis with modified Niskin^®^ bottles from 4 to 5 depths at three locations in 2000 off Long Island, NS, with bottom depths ranging from 83m near shore to 180m over the Grand Manan Basin. The program was expanded in 2001 and 2002 to collect seawater from 5 locations and six depths spaced across the entrance of the Bay of Fundy, which samples the easterly inflow at the surface from the Scotian Shelf and at depth from the Northeast Channel and the outflow at two stations in the Grand Manan Channel (Fig 1, S1 Table). Unfiltered seawater samples were collected for both MeHg and THg determinations using a General Oceanics Lever Action Niskin modified for trace metal sampling. The Niskin modification involved Teflon end-caps, drain spout and internal coating. To further reduce the possibility of contamination, established “clean sampling methods” were employed with the sub-sampling carried out in a clean area of boat. Seawater for mercury determinations was drawn first from the Niskin. The water samples were collected into pre-cleaned Teflon bottles and double bagged until the samples could be preserved. Total Hg samples were preserved with 2 ml/L BrCl and MeHg samples were preserved with 2 ml/L 9M H_2_SO_4_. The “pickling” step was usually carried out within 2 hours of collection when the boat became stable or at dockside. Water samples for salinity and nutrients were also collected at each sampling. This salinity data was compared to a Seabird^®^-CTD profile to confirm our Niskin sampling depths, especially in the deeper waters of the Northeast Channel.

All larger organisms were collected individually in the field where specimens were also carefully placed in polyethylene zip bags and stored at -20°C in the lab until analysis (S4 and S5 Tables). Two rockweed species, bladder wrack (*Fucus vesiculosus*) and knotted wrack (*Ascophyllum nodosum*), were collected by hand from shore in Specht’s Cove, NS in October, 2003. Three sets of 10 blue mussels (*Mytilus edulis)* were collected from shore at each of the Specht’s Cove, Digby Harbour and Apple River, NS, locations around the bay in October 2003. Ten American lobsters (*Homarus americanus*) and 9 sea scallops (*Placopecten magellanicus*) were collected in 2000 from near Grand Manan.

Fishes were collected by the Department of Fisheries and Oceans (DFO), Canada, groundfish survey in the Bay of Fundy and the large pelagics observer program or as donations from Digby Neck fishermen (S4 Table). These fish were variously caught by handline, longline or bottom trawls between 2001 and 2002. Twenty Atlantic cod (*Gadus morhua*), 24 haddock (*Melanogrammus aeglefinus*), 10 pollock (*Pollachius virens*), 8 white hake (*Urophycis tenuis*), 9 cunner (*Tautogolabrus adspersus*), 15 Atlantic herring (*Clupea harengus*), 14 Atlantic mackerel (*Scomber scombrus*), 14 winter flounder (*Pseudopleuronectes americanus*), 14 yellowtail flounder (*Limanda ferruginea*), 16 spiny dogfish (*Squalus acanthias*), 1 common thresher shark (*Alopias vulpinus*), 5 Atlantic bluefin tuna (*Thunnus thynnus*) and 11 swordfish (*Xiphias gladius*) were collected. Swordfish, shark and tuna tissues were subsampled at sea with a stainless steel knife for liver, muscle and fat tissues and the tissues frozen individually.

Marine mammals stranded near our study area were reported to us by local DFO fisheries officers, which enabled us to collect blubber and muscle tissue. Additional samples were made available from the Gulf of Maine and the Grand Manan area from the Cape Cod Stranding Network, Inc. and the Grand Manan Whale and Seabird Research Station Ltd. In all 3 minke whales (*Balaenoptera acutorostrata*), 3 fin whales (*Balaenoptera physalus*), 3 humpback whales (*Megaptera novaeangliae*), 3 Atlantic white-sided dolphin (*Lagenorhynchus acutus*), and 10 harbour porpoise (*Phocoena phocoena*) were obtained from stranding events or net drowning between 2000 and 2003 (S5 Table). As with the large pelagic fish, marine mammals were subsampled for liver, muscle and fat or blubber tissues at the stranding site and stored frozen in polyethylene zip bags.

#### Ethics statement

Marine fish surveys conducted at sea present a special set of conditions with respect to euthanasia. Guidelines developed by the American Fisheries Society state that fish collected in this way can be exempted from standard practices of euthanasia due to the numbers of specimens collected at one time (https://fisheries.org/policy-media/science-guidelines/guidelines-for-the-use-of-fishes-in-research/#8.1). Invertebrates and fish species died either through the method of capture, on deck, or by rapid freezing. The Animal Care Committee of Fisheries and Oceans, Canada, Maritime Region, approved these methods of euthanization and the study was carried out under the auspices of Fisheries and Oceans, Canada. Marine mammals were sampled from dead individuals that had stranded and the immediate cause of their death is unknown but not as a result of this study. Samples collected in the Bay of Fundy were done so with authorization to engage in fishing and related activities on the Atlantic coast of Canada subject to the provisions of the Fisheries Act and Regulations bestowed by the Regional Director of Science, Science Branch, Maritimes Region, Dartmouth, Nova Scotia, Canada. No specific permissions were required for other samples (seaweed, plankton) and the field studies did not involve endangered or protected species.

### Sample processing

All samples were processed on shore in a chemistry laboratory used for mercury analysis. Chalk-free plastic gloves were worn during all phases of sample handling. Frozen bags of plankton samples to be processed each day were thawed first thing in the morning in a container of high purity water for 30 to 45 minutes. It was found sufficient to homogenize thawed plankton samples of size fractions between 25 to 250μm by hand for 20 to 30 seconds in their original sample bags. The 500μm to 4mm size fractions were homogenized in their sample bags using a Polytron^®^ Brinkman Homogenizer probe. The 8 to 16mm plankton size fractions were removed from their sample bags and homogenized in a Cuisinart^®^ Blender. The polycarbonate bowl and stainless steel blade from the blender were wiped clean with lab wipes and washed with high purity water (Millipore Super Q ^®^) between samples. After the homogenization of each plankton sample, a 10g aliquot of the mixture was placed in a Bitran ^®^ 3mil polyethylene zipper bag, double bagged and stored at -20°C. Seaweed, shucked mussels and scallops, shelled lobsters, entire fish and selected muscle, liver and fatty tissues of large pelagic fish and marine mammals were blended to a paste with a variety of commercial or domestic food processors (plastic with stainless steel blades), depending on the size of organism. Food processors and utensils were washed with soap and water and rinsed with distilled water (Millipore Super Q^®^) between samples. After the completion of all sample processing, the frozen 10g subsamples were packed in dry ice and shipped via air freight to Flett Research Ltd. (Winnipeg, MB, Canada) for MeHg and THg analysis.

Sample aliquots were dried for 48 hours at 60°C in both aluminum pans and scintillation vials for determining wet weight to dry weight conversions and naturally occurring stable N and C isotopes, respectively. Selected vials of measured (~1mg) dried, powdered tissue were encapsulated in a tin cup and sent to the Department of Soil Science, University of Saskatchewan (Saskatoon, SK, Canada) for isotope analysis.

### Stable isotope analysis

Tin capsules containing tissues were combusted at 1800°C in a Robo-Prep elemental analyzer for stable isotope determinations. Evolved CO_2_ and N_2_ gases were analysed using an interfaced Europa 20:20 continuous-flow isotope ratio mass spectrometer (CFIRMS). Albumen standards were spaced after every fifth tissue analysis. Stable isotope concentrations were expressed in δ notation as the deviation from standards in parts per thousand (‰) according to the following equation:

δX=[(Rsample/Rstandard)−1]*1000

where X is ^13^C or ^15^N and R is the corresponding ratio of ^13^C/^12^C or ^15^N/^14^N. Samples depleted in the heavier isotopes, either ^13^C or ^15^N, in comparison to the standard, have lower δ values. The R_standard_ values were based on the PeeDee Belemnite for ^13^C/^12^C and atmospheric nitrogen for ^15^N/^14^N. Replicate measurements of internal laboratory standards indicate errors of ±0.1‰ and ±0.3‰ for δ^13^C and δ^15^N, respectively [43].

### Trophic level calculations

The heavier N^15^ isotope is retained, relative to N^14^, during food consumption resulting in an enrichment of δ^15^ N between 3 to 5‰ increments per trophic level [29, 44,45]. A value of 3.4 was adopted for the present study following a review of the literature on δ^15^N trophic increments by Post [46]. The average δ^15^N value of our smallest size fraction sampled (25μm) was -0.03, which we used as the base of our trophic food web. Trophic level (TL) can then be calculated as:

TLorganism=1+(δ15Norganism+0.03)/3.4.

Stable carbon isotopes are less useful for trophic level analysis because there is less than 1% enrichment of δ^13^C per trophic level [29] but this stability may enable inference on the relative importance of benthic-based versus planktonic-based food chains [47].

Biological studies on the planktonic-pelagic size spectrum in lakes and oceans have shown that three log_2_ size units generally represent the differences in size between prey and predator (e.g. phytoplankton (25μm) to mesoplankton (250μm)) [30,32,48]. As stated above, our net tows were sorted through a Wentworth sieve series to enable comparison of the size spectra to the isotope approach of deriving trophic levels. Sizes of the larger organisms collected were standardized as estimated spherical diameters (ESD). Individual fish, benthic invertebrate and marine mammal ESDs were estimated from their wet weight assuming a spherical shape with a density of 1g.cm^-3^. Some large fish, such as swordfish and common thresher shark, had their wet weight first estimated from length measurements [49,50] before ESD could be calculated (S4 Table). Atlantic bluefin tuna had a rounded weight measured in kg from the vendor at the wharf. Humpback, minke and fin whale weights were calculated from length measurements using weight-length equations given by Lockyer [51] (S5 Table). All of the dolphins and all but one of the autopsied porpoises were weighed in the field. The length-weight equation of Read and Tolley [52] was used to approximate fresh weight of the remaining porpoise to calculate ESD.

### Bioaccumulation metrics

Bioconcentration factors (BCF) are used to quantify the difference between the concentrations of mercury in primary producers (ng/g wet weight) and the concentration in seawater (pg/L seawater):

BCF=[organism]/[seawater].

Biomagnification factors (BMF) are used to quantify the difference between mercury concentrations (ng/g wet weight) at consecutive trophic levels determined either by the δ^15^N or size method:

BMF=[TLx+1]/[TLx].

It is known that mercury concentrations in marine organisms increase exponentially with organism size, as a power function. The resulting regression is lognormal and a total magnification factor (TMF) can be calculated as the antilog of the slope, b, of the equation:

$$[Mercury]=10^{bTL}$$

such that

Log10[Mercury]=a+bTL

and

$$TMF=10^{b}.$$

This index was previously used as a measure of trophic magnification [53]; however, it is not known precisely how mercury was acquired by organisms in nature from field sampling. Mercury can be incorporated into an organism by ad- and absorption from its environment, through prey consumption and initially from female to offspring transfer.

### Mercury analysis

#### Seawater

The water samples were analysed in a dedicated mercury laboratory at the Bedford Institute of Oceanography (Dartmouth, NS, Canada) using US EPA methods 1631 for THg [54,55] and EPA method 1630 for MeHg [56]. Prior to total Hg analysis, samples were digested at 60°C for 24 hours and if excess BrCl was not evident in the sample, additional BrCl was added and the heat-digestion step repeated. The MeHg samples were stored at -4 °C prior to analyses. A certified reference standard (ORMS-2 for THg and DORM-2 for MeHg) was carried out to determine the accuracy of these methods. The analysis of a MeHg certified reference standard, supplied by Brooks-Rand (Seattle, WA), was also used as a check of the accuracy of the MeHg method. Recovery of THg from seawater averaged 103±10% (N = 12). The minimum detection level, measured as three standard deviations above blank values, was 40 pg/L (N = 18) for THg and 7 pg/L (n = 6) for MeHg. Precision, measured as the percentage relative difference, (standard deviation/mean)*100, of paired duplicates averaged 6.9% (n = 4) for THg and 8.2% (n = 4) for MeHg.

### Biota

All tissue samples were previously homogenized, allowed to thaw and then re-homogenized with a clean stainless-steel spatula. Approximately 200mg was removed and weighed into acid cleaned test tubes. As well as the tissue samples, there were two duplicate samples, two sample spikes, two analytical blanks, two test tubes with Dorm-2 or Dolt-2 and four with the F.W.I. Mercury Quality Assurance Program (MQAP) reference material for each thirty test tubes run. 10ml of 1:2.5 nitric/sulfuric acid mixture was added to each tube and heated at 180°C for 6 hours in an aluminum hot block. After cooling, the sample volumes were brought up to 20ml with low mercury deionized water and 200μl of BrCl was added. The contents of the test tubes were quantitatively transferred to 40ml acid cleaned EPA vials and the final volumes brought up to 25ml, again with low mercury deionized water. Aliquots ranging from 100 to 1000μl were drawn from this final solution for analysis.

Hydroxylamine hydrochloride was added to whale blubber samples to destroy residual BrCl and stannous chloride was used to reduce the mercury. The elemental mercury produced was bubbled off and collected on gold traps. The traps were then heated in an argon gas stream and the mercury released was measured by atomic fluorescence spectroscopy [55, 57]. The detection limit was about 0.5ng Hg/g wet weight.

Approximately 200mg of homogenized tissue sample was removed and weighed into acid cleaned 22ml Teflon vials for MeHg analysis. Besides the samples, there were two sample spikes, a sample duplicate, two analytical blanks and two vials with either Dorm-2 (for tissue, muscle or fat) or Dolt-2 (for liver). 1.5ml of 25% KOH in MeOH was added to each vial, and the vials were tightly capped and digested at 75°C overnight. After cooling, either 5ml or 20ml of MeOH were added to each plankton or fish/marine mammal vial, respectively. Aliquots ranging from 30 to 60μl were drawn from this final solution for analysis. Sodium tetraethyl borate was used to ethylate the methyl mercury to ethylmethyl mercury, which was purged onto a Tenax trap and dried with nitrogen. The trap was heated in an argon gas stream, which delivered the analyte to a GC column for separation of the ethylmethyl mercury from other ethylated mercury compounds [58]. The analytes were passed through a pyrolizer where the organic mercury was converted to Hg^0^ before entering a cold vapour atomic fluorescence analyzer for quantification [59]. The detection limit was about 0.5ng MeHg/g wet weight. Spike and recoveries typically were 100±10%. Precision estimates, as measured on paired duplicates of swordfish tissue for example, were ±2.6% (N = 8).

### Statistical analysis

Parametric (t-test, ANOVA, ANCOVA) and nonparametric (Kruskal-Wallis) statistics were used throughout, using a significance level of P < 0.05 with SYSTAT^®^ 5.2 or Mac. Regressions were calculated to best fit our observed distributions with DataGraph^®^ version 4.2.

## Results and discussion

### Seawater

Mercury measured in unfiltered seawater collected during late spring and summer between 2000 and 2002 (Fig 1), showed that neither MeHg nor THg concentrations were significantly different between the various years or depths (upper 25m, 30 to 100m and >100m) sampled (ANOVA, Table 1). The calculated flux of THg in seawater across the mouth of the Bay of Fundy along our transect was balanced [60]. MeHg concentrations ranged from 10 to 99 pg/L with a median value of 56 pg/L (N = 55). Total mercury concentrations ranged from 17 to 548 pg/L with a median value of 237 pg/L (N = 69).

**Table 1:** Mercury concentrations in bulk seawater between 2000 and 2002, from the approaches to the Bay of Fundy, Gulf of Maine.

Date	Depth (m)	n	MeHg (pg/L) X±SD	MeHg (pg/L) Median (range)	n	THg (pg/L) X±SD	THg (pg/L) Median (range)
21-24/08/00	0–25				5	256±55	272 (200–324)
	>25–100				4	264±89	267 (179–345)
	>100				8	223±44	207 (177–304)
	All depths				17	242±59	219 (177–345)
11-21/06/01	0–25	12	49.9±20.6	55.5 (10.7–73.6)	12	260±27	258 (221–310)
	>25–100	12	52.5±25.0	47.0 (12.1–99.1)	12	275±125	236 (138–548)
	>100	4	63.4±23.7	61.4 (40–90)	4	187±133	216 (17–298)
	All depths	28	53.0±22.6	50.5 (10.7–99.1)	28	256±98	253 (17–548)
26/08-05/09/02	0–25	10	55.7±20.8	52.6 (23.5–84.3)	8	221±45	221 (159–291)
	>25–100	13	68.4±19.3	72.5 (32.9–90.3)	12	230±56	239 (119–310)
	>100	4	74.8±28.1	85.3 (33.4–95.2)	4	199±39	194 (158–249)
	All depths	27	64.0±21.4	70.7 (23.5–95.2)	24	222±50	226 (119–310)
All dates	All depths	55	58.4±22.5	56.1 (10.7–99.1)	69	237±74	236 (17–548)

Total mercury values are the same order-of-magnitude as previous measurements taken in neighbouring regions of the Gulf of St. Lawrence (Mean of 485 pg THg /L [61]) and on the Scotian Shelf (242–686 pg THg /L [62]), both sampled in 1985. North Atlantic central waters had lower mercury values of 131±64 pg THg/L in 2010 [63]. Bay of Fundy THg concentrations are considerably below levels found in contaminated, coastal areas such as New York/New Jersey Harbor, USA, in 2002–2003 (3.5–65.9 ng THg /L [64]). Methylmercury levels reported here are above the levels reported for the central North Atlantic surface waters (12.1±10.1) [63] but within the range measured in open ocean areas such as in the Arctic Ocean (57–95 pg MeHg /L [65]), Mediterranean Sea (10–100 pg MeHg /L [66]) and North Sea (16–64 pg MeHg /L [67]) but less than Newark Bay, USA, (40–360 pg MeHg /L [64]). Mercury concentrations measured here in seawater are several orders-of-magnitude below sublethal effects reported for either phytoplankton [68, 69] or zooplankton [70,71].

### Plankton/nekton

**Fig 2:**
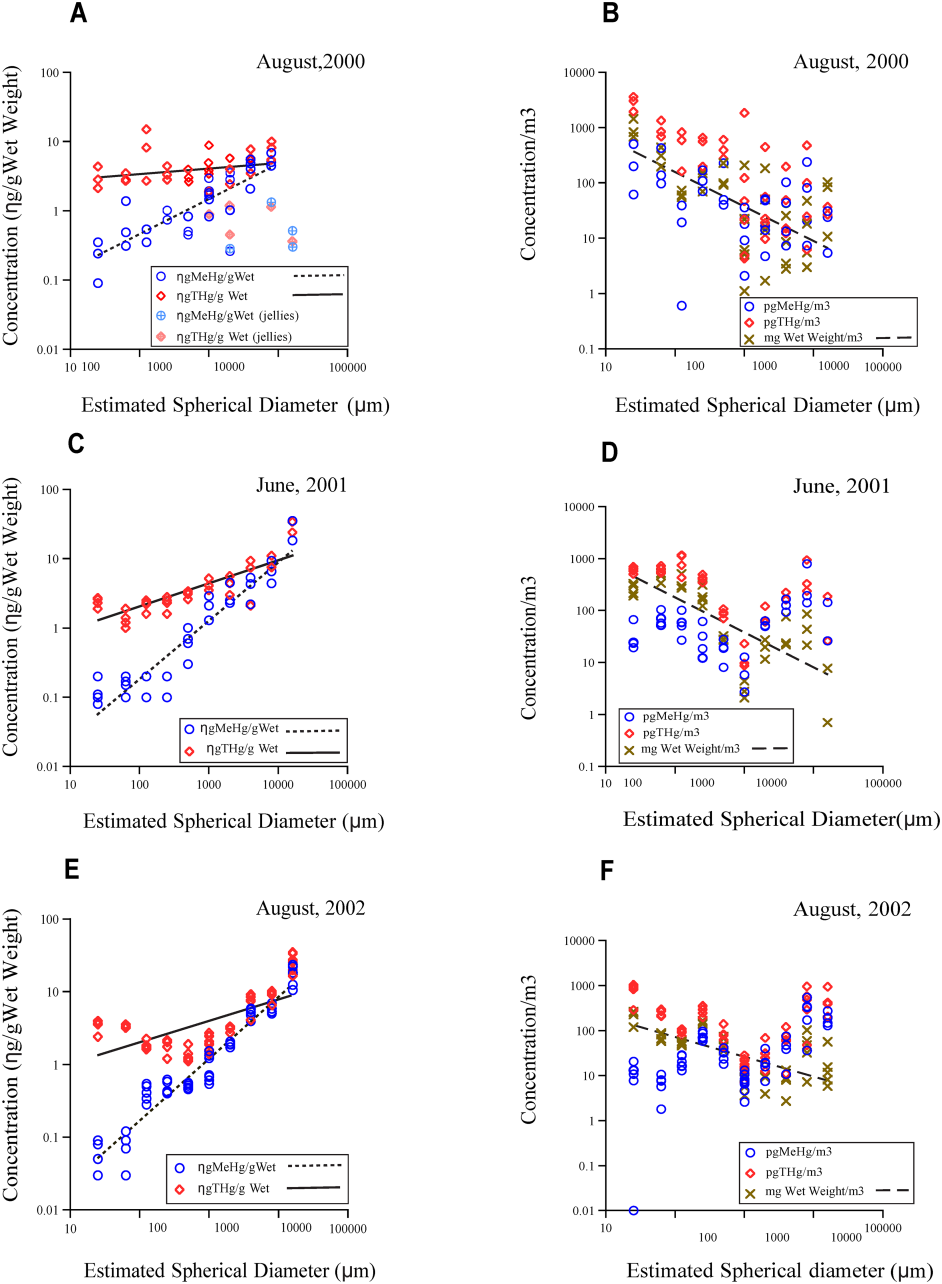
The relationship between MeHg and THg concentrations on both an organism (ng size category/g wet weight) and a volume (pg size category/m^3^ seawater) basis plotted against organism size (ESD). Also illustrated on a volume basis is biomass (mg wet weight/m^3^ seawater) versus ESD.

Ten size categories of organisms were sampled to document the transition between predominately surface ad/absorption to trophic uptake of mercury in the lower trophic levels of the pelagic ecosystem (Fig 2). The log_2_ sieve series chosen had the added advantage of concisely sorting the catch to species and/or developmental stage, such that each screen contained at most three dominant taxa and a maximum of five, if common taxa are included (Table 2). Diatoms dominated the phytoplankton in microplankton categories from the late spring to late summer sampling periods. Strong tidal mixing of the water column in the area is not conducive to summer stratification of the surface waters. A dinoflagellate flora, some of which ingest prey, consequently was not present contrary to the seasonal succession found elsewhere in the Gulf following the spring bloom [72].

**Table 2:** Taxonomic composition of planktonic to pelagic size groupings.

Size range	Category	Dominant Taxa	Common Taxa
25–63μm	Microplankton (~90%)*	*Pleurosigma* spp.	*Dictyocha* spp.
		*Thalassionema nitzschioides*	*Melosira nummuloides*
63–125μm	Microplankton (~92%)*	*Streptotheca* spp.	*Ceratium tripos*
		*Rhizosolenia alata*	Copepod nauplii
125–250μm	Microplankton (~54%)*	*Rhizosolenia alata*	*Coscinodiscus* spp.
		*Oithona* spp.	*Pleurosigma* spp.
		copepodites	
250–500μm	Mesoplankton	*Calanus* copepodites	*Acartia hudsonica*
		*Pseudocalanus* spp.	*Centopages* spp.
		copepodites	*Temora longicornus*
500–1000μm	Mesoplankton	*Calanus finmarchicus* copepodites	*Centopages* spp.
		*Pseudocalanus* spp. adults	*Temora longicornus*
1-2mm	Macroplankton	*Calanus finmarchicus* adults	*Thysanoessa* spp.
		*Limacina retroversa*	*Anomalocera opalus*
2-4mm	Macroplankton	*Calanus hyperboreus*	*Thysanoessa inermis*
		*Themisto* spp.	*Limacina retroversa*
		*Euchaeta norvegica*	
4-8mm	Nekton	*Meganyctiphanes norvegica*	*Pleurobrachia pileus*
		*Themisto compressa*	*Thysanoessa inermis*
			*Clupea harengus* larvae
8-16mm	Nekton	*Meganyctiphanes norvegica*	*Mitrocomella polydiademata*
		*Pleurobrachia pileus*	*Cyanea capillata*
>16mm	Nekton	*Pasiphaea multidentata*	*Mitrocomella polydiademata*
			*Cyanea capillata*

* Percentage phytoplankton by count.

Concentrations of THg and MeHg over the three years sampled in our coastal ecosystem were lowest in the phytoplankton-dominated categories and gradually increased with size from the zooplankton to nektonic size categories (Fig 2, Table 3). THg concentrations across the micro- and mesoplankton size range (25 to 500μm) were relatively level in 2000 and 2001 but declined slightly with size in 2002 (Fig 2). MeHg concentrations increased more or less continuously in 2000 and 2001, whereas the 125μm and 250μm size fraction concentrations in 2002 remained at the 63μm levels before increasing to the nekton categories.

**Table 3:** Methylmercury and total mercury concentrations (wet weight basis) in selected categories that represent the food web located at the approaches to the Bay of Fundy, Gulf of Maine.

Species	Weight (gm) Median (range)	MeHg (ng/g) X±SD	MeHg (ng/g) Median (range)	THg (ng/g) X±SD	THg (ng/g) Median (range)	n
Phytoplankton (25–63μm) (~90%)*		0.12±0.10	0.09 (<0.1–0.35)	3.0±0.8	2.8(1.9–4.3)	12
Phytoplankton (63–125μm) (~92%)*		0.27±0.37	0.14 (0.03–1.38)	2.6±0.9	3.0(1.0–3.5)	12
Phyto-/zooplankton (125–250μm) (~54%)*		0.30±0.18	0.31 (<0.1–0.54)	3.6±4.0	2.2(1.6–8.2)	12
Mesoplankton (250–500μm)		0.48±0.32	0.42 (0.1–1.0)	2.4±0.9	2.2(1.2–4.4)	12
Mesoplankton (500–1000μm)		0.58±0.19	0.53 (0.3–1.0)	2.4±1.0	2.6(1.1–4.0)	12
Macroplankton (1.0–2.0mm)		1.37±0.72	1.37 (0.54–2.9)	3.1±1.8	2.5(1.4–8.8)	18
Macroplankton (2.0–4.0mm)		2.1±1.18	1.9 (0.26–2.8)	3.3±1.5	3.2(0.5–5.8)	13
Nekton (4.0–8.0mm)		4.45±1.23	4.8 (2.1–5.9)	6.5±2.3	7.4(2.1–9.4)	12
Nekton (8.0–16.0mm)		5.5±1.9	5.4 (1.3–9.3)	8.3±2.8	9.2(1.1–11.0)	12
Nekton (>16.0mm)		14.5±11.1	17.3 (0.3–35.1)	26.9±6.8	26.4(16.6–34.7)	8
Knotted wrack ^b^ (*Ascophyllum*)	255	0.16±0.06	0.2 (0.1–0.2)	12.0±1.0	11.8(11.2–13.1)	3
Bladder wrack ^b^ (*Fucus*)	170	0.2	0.2	6.2±0.2	6.2(6.0–6.3)	3
Blue mussel ^b^ (*Mytilus*)	5.2 (3.7–7.8)	5.3±1.1	5.1 (3.7–7.1)	19.6±5.6	20.1(10–27.3)	12
American lobster ^b^ (*Homarus*)	349 (266–386)	27.1±11.1	24.1 (17.2–47.9)	35.9±10.2	33.5(23–55)	9
Glass shrimp ^b^ (*Pasiphaea*)	(3–5)	19.7±7.6	18.4 (10.6–35.1)	27.1±7.0	26.4(16.6–35.1)	8
Pollock ^d^ (*Pollachius*)	135.5 (101.9–1368.0)	15.4±5.4	14.2 (8.9–24.3)	18.7±7.1	15.6 (10.4–32.0)	10
Cunner ^d^ (*Tautogolabrus*)	102.9 (60.6–186.3)	75.3±28.1	75.1 (34.4–122.1)	79.7±27.9	84.9 (39.9–122.1)	9
Atlantic mackerel ^p^ (*Scomber*)	146 (82–349.4)	17.4±5.3	16.5 (10.2–28.5)	21.8±6.0	21.5 (12.5–30.0)	14
Atlantic herring ^p^ (*Clupea*)	160.9 (58–206.6)	40.3±25.2	38.0 (5.5–79.6)	47.5±28.4	41.6 (9.2–98.0)	19
Haddock ^d^ (*Melanogrammus*)	580.8 (233.8–1754.3)	18.3±11.5	14.5 (7.0–48.0)	32.3±14.1	26.7 (19.0–68.9)	16
Atlantic cod ^d^ (*Gadus*)	952.9 (583.5–4091.0)	27.1±14.0	26.0 (10.0–52.0)	35.2±16.0	28.0 (19.7–79.2)	19
White hake ^p^ (*Urophycis*)	1009.5 (617–1942.3)	24.1±9.8	25.4 (11.0–42.2)	29.5±6.6	27.5 (23.0–43.9)	8
Winter flounder ^b^ (*Pseudopleuronectes*)	218.5 (151–361.6)	15.2±7.8	15.0 (3.2–28.0)	21.1±8.1	17.5 (9.4–32.7)	14
Yellowtail flounder ^b^ (*Limanda*)	520.9 (361.5–1027.1)	23.3±8.7	22.4 (9.9–47.3)	26.9±9.7	25.0 (12–50.7)	14
Spiny dogfish ^p^ (*Squalus*)	1347 (691–3241)	83.9±27.0	79.9 (4.4–156.2)	99.3±27.2	103.2 (63.6–169.2)	16
Swordfish ^p^ ^e^ (*Xiphias*)	103900 (56400–148500)	639±229	628 (249–1187)	1245±729	1192 (428–3233)	11
Bluefin tuna ^p^ ^e^ (*Thunnus*)	323000 (313000–379000)	496±102	504 (397–639)	565±88	576 (465–696)	5
Thresher shark ^p^ ^e^ (*Alopias*)	561400	1427		1472		1
Harbour porpoise ^e^ (*Phocoena*)	28.4 (12.1–47.2)	325.8±230.1	253.3 (49.1–748.0)	606.8±679.4	288.8 (129.7–2307.6)	10
White-sided dolphin ^e^ (*Lagenorhynchus*)	158.7 (127.0–183.7)	510.8±52.5	497 (466.6–568.8)	1261.4±116.2	1262.0 (1144.5–1376.9)	3
Minke whale ^e^ (*Balaenoptera*)	3741.8 (809–8223)	72.5±71.7	70.8 (1.2–165.2)	79.4±73.7	84.7 (2.7–171.1)	5
Humpback whale ^e^ (*Megaptera*)	5685.5 (4604–19762)	32.2±53.2	1.9 (1.1–93.7)	43.3±63.8	7.3 (5.6–117.0)	3
Fin Whale ^e^ (*Balaenoptera*)	33145.8 (31656–97032)	6.6±4.1	8.9 (1.9–9.1)	26.0±16.6	17.0 (15.8–45.1)	3

* percentage phytoplankton.

^d^ demersal.

^p^ pelagic.

^b^ benthic.

^e^ whole organism mercury levels extrapolated from selected tissue levels.

There were few differences found for mercury levels within individual size categories between sampling years: 25μm fractions, 2002 > 2001, P<0.05; 63μm fractions, 2001 and 2002> 2000, P<0.05; 125μm and 250μm fractions, 2000 > 2001 and 2002, P < 0.05; 500μm fractions, 2000 and 2001> 2002; 1mm fraction, 2000 and 2001> 2002, P < 0.05 and 16mm fraction, 2001 and 2002> 2000, P < 0.001 (Kruskal-Wallis and t-tests). The low MeHg and THg concentrations between 4 and 16mm nektonic categories in 2000 were associated with the dominance of jelly-like organisms (ephyra stages of *Cyanea*, hydromedusae and the sea gooseberry, *Pleurobrachia*), and their greater proportion of water to carbon content (Fig 2). The anomalously low 16mm-size mercury values in the 2000 samples are indicated separately in Fig 2 and were deleted from the best fit equations:

$$\left[\text{MeHg}\right]\;\text{versus}\;\text{size}\;\left(\text{ESD}\right);\;\text{Y}=0.083{\text{X}}^{0.\text{48}\pm 0.08},{\;\text{r}}^{2}=0.43,\;\text{n}=34\;\left(\text{Panel}\;\text{A}:\;\text{August},2000\right);$$

$$\left[\text{MeHg}\right]\;\text{versus}\;\text{ESD},\text{Y}=0.004{\text{X}}^{\text{0}.\text{84}\pm \text{0}.\text{07}},{\;\text{r}}^{2}=0.88,\text{n}=34\;\left(\text{Panel}\;\text{C}:\;\text{June},2001\right);$$

$$\left[\text{THg}\right]\;\text{versus}\;\text{ESD},\text{Y}=0.44{\text{X}}^{\text{0}.\text{33}\pm \text{0}.\text{04}},{\text{r}}^{2}=0.69,\text{n}=34\;\left(\text{Panel}\;\text{C}:\;\text{June},2001\right);$$

$$\left[\text{MeHg}\right]\;\text{versus}\;\text{ESD},\text{Y}=0.003{\text{X}}^{\text{0}.\text{87}\pm \text{0}.\text{03}},{\text{r}}^{2}=0.97,\text{n}=55\;\left(\text{Panel}\;\text{E}:\;\text{August}\;2002\right);$$

$$\left[\text{THg}\right]\;\text{versus}\;\text{ESD},\text{Y}=0.52{\text{X}}^{\text{0}.\text{30}\pm \text{0}.\text{04}},{\text{r}}^{2}=0.43,\text{n}=55\;\left(\text{Panel}\;\text{E}:\;\text{August}\;2002\right).$$

A comparison of the mercury concentrations, as ng/g wet weight, in different size categories of plankton/nekton and sampling years indicates the effect of year (2000, 2001, 2002) and the covariant organism size and the interaction between year and size were significant for both MeHg and THg (ANCOVA, P < 0.001, n = 126). A further test of heterogeneity for the fitted regressions in Fig 2 confirmed that the slopes for MeHg in 2001 and 2002 were similar (P < 0.001). Methylmercury concentrations of whole body tissue in the 10 size categories of plankton /nekton sampled (ng/g wet weight) increased more steeply than THg concentrations in all three years. There was no discernable trend in plankton /nekton water column mercury concentrations (pg plankters/m^3^) with the possible exception of 2000 (Fig 2). In August 2000, the THg and MeHg water column concentrations of macrozooplankton and nekton (pg plankton or nekton/m^3^ of seawater) were variable and lower than in the micro- and mesoplankton. As stated, the larger nektonic categories in 2000 contained gelatinous species, which could explain the drop in mercury concentrations on a wet weight basis and this resulted in lower and more variable pg/m^3^ concentrations (Fig 2).

Biomass of plankton and nekton categories in the water column (mg wet weight/m^3^) decreased, as expected, with increasing size of organism in all years (Fig 2):

$$\text{Biomass}\;\left(\text{mg}/{\text{m}}^{3}\right)\;\text{versus}\;\text{ESD},\text{Y}=7649{\text{X}}^{-\text{0}.\text{81}},{\text{r}}^{2}=0.60,\text{n}=34\;\left(\text{Panel}\;\text{B}:\;\text{August},2000\right);$$

$$\text{Biomass}\;\left(\text{mg}/{\text{m}}^{3}\right)\;\text{versus}\;\text{ESD},\text{Y}=3916{\text{X}}^{-\text{0}.\text{67}},{\text{r}}^{2}=0.55,\text{n}=34\;\left(\text{Panel}\;\text{D}:\;\text{June},2001\right);$$

$$\text{Biomass}\;\left(\text{mg}/{\text{m}}^{3}\right)\;\text{versus}\;\text{ESD},\text{Y}=544{\text{X}}^{-\text{0}.\text{44}},{\text{r}}^{2}=0.49,\text{n}=54\;\left(\text{Panel}\;\text{F}:\;\text{August}\;2002\right).$$

The ten plankton to nekton size fractions, for the three years sampled combined, had THg concentrations (ng/g wet weight) that increased from 3.0±0.8 in the smallest fraction (25μm) to 26.9±6.8 in the 16mm fraction (Table 3). Similarly, MeHg concentrations increased from 0.12±0.10 in the 25μm fraction to 14.5±11.1 ng/g wet weight in the 16mm fraction. The Bay of Fundy mesoplankton THg values (2.4±0.9 ng/g wet weight) are comparable in value to similar-sized plankton from Hudson Bay (2.6±0.2ng) [73], Gulf of St. Lawrence (2.5±0.1ng) [25] and the Mid Atlantic Bight (3.7±3.7) [74] (Table 3). Concentrations in the macroplankton/nekton categories reported here as predominately euphausiids (8.3±2.8 ngTHg wet weight) are similar to values found in the Greenland Sea at 26±8 [75], Hudson Bay at 4.7±0.8ng [73] and the Gulf of St. Lawrence at 12.0 ±0.9ng [25]. The plankton and nekton mercury levels reported here, therefore, appear to be similar to values reported over a broad latitudinal range from the subarctic to temperate ecozones.

### Other biota

Two seaweeds, bladder and knotted wracks, collected had mercury levels in the low ppb range (Table 3; 6–13 ng/g wet weight). No mercury measurements are available, to the best of our knowledge, for the western N. Atlantic but comparable levels have been documented for similar species in Norwegian fjords [76] and along the coastlines of the North and Baltic Seas [77].

The blue mussel has median mercury levels of 20 (10–27) ng THg/g wet weight off SW Nova Scotia (Table 3), which is consistent with other reports from the northern Gulf of Maine between 2003–2008 [35]. Sea scallops had similar mercury levels to the mussels (Table 3) and sea scallops near Sable Island, N.S., [78] and Passamaquoddy Bay, NB [79]. Mercury levels in American lobster (Table 3) caught off Grand Manan, at the entrance to the Bay of Fundy, were an order-of-magnitude lower than those analysed in the southern Gulf of Maine [80].

Most fish collected for this food chain study were analysed whole for mercury, purposely for food chain interpretations, whereas the values reported for these species in the literature are for those tissues used for human consumption. Thus, most of the levels reported here (Table 3) are not directly comparable to specific tissue levels given in the literature. It was not feasible to analyse entire swordfish, Atlantic bluefin tuna, common thresher shark and marine mammals which enables tissue comparisons with previous reports (S4 Table and S1 Text).

### Food web descriptors

Carbon and nitrogen stable isotopes are used to gain insight into trophic functioning of aquatic food webs [29,45] and how this relates to the biomagnification of mercury [22,81–83].

The enrichment of the heavy carbon isotope ^13^C relative to ^12^C in plants, expressed as δ^13^ C, is related to the phylogenetic type of plant (photosynthetic route) at the base of the food chain [29, 84]. δ^13^ C changes minimally between trophic levels (0.2 to 1‰), which is thought to be potentially useful in determining carbon source at various levels of the food chain [85–87]. In the present study, however, the phytoplankton δ^13^ C values of -16.2±1.2 ‰ for the 25um fraction were indistinguishable from the rockweed *Ascophyllum* (-16.1±0.4‰)(see S2 Text for a fuller discussion of carbon isotope ratios for other primary producers in the Bay of Fundy).

Prouse et al. [88] estimated that 96% of primary production in the Bay of Fundy was produced by phytoplankton, 2% by macroalgae beds, 1% by benthic microalgae and 0.6% from salt marsh grasses. The limited area available for primary producers other than phytoplankton in the Bay of Fundy suggests that a predominantly planktonic energy source supports the benthic, demersal and pelagic organisms in the Bay of Fundy and adjacent Gulf of Maine. In general, few studies have been able to distinguish separate food webs from benthic or pelagic δ^13^ C food sources [53].

**Fig 3:**
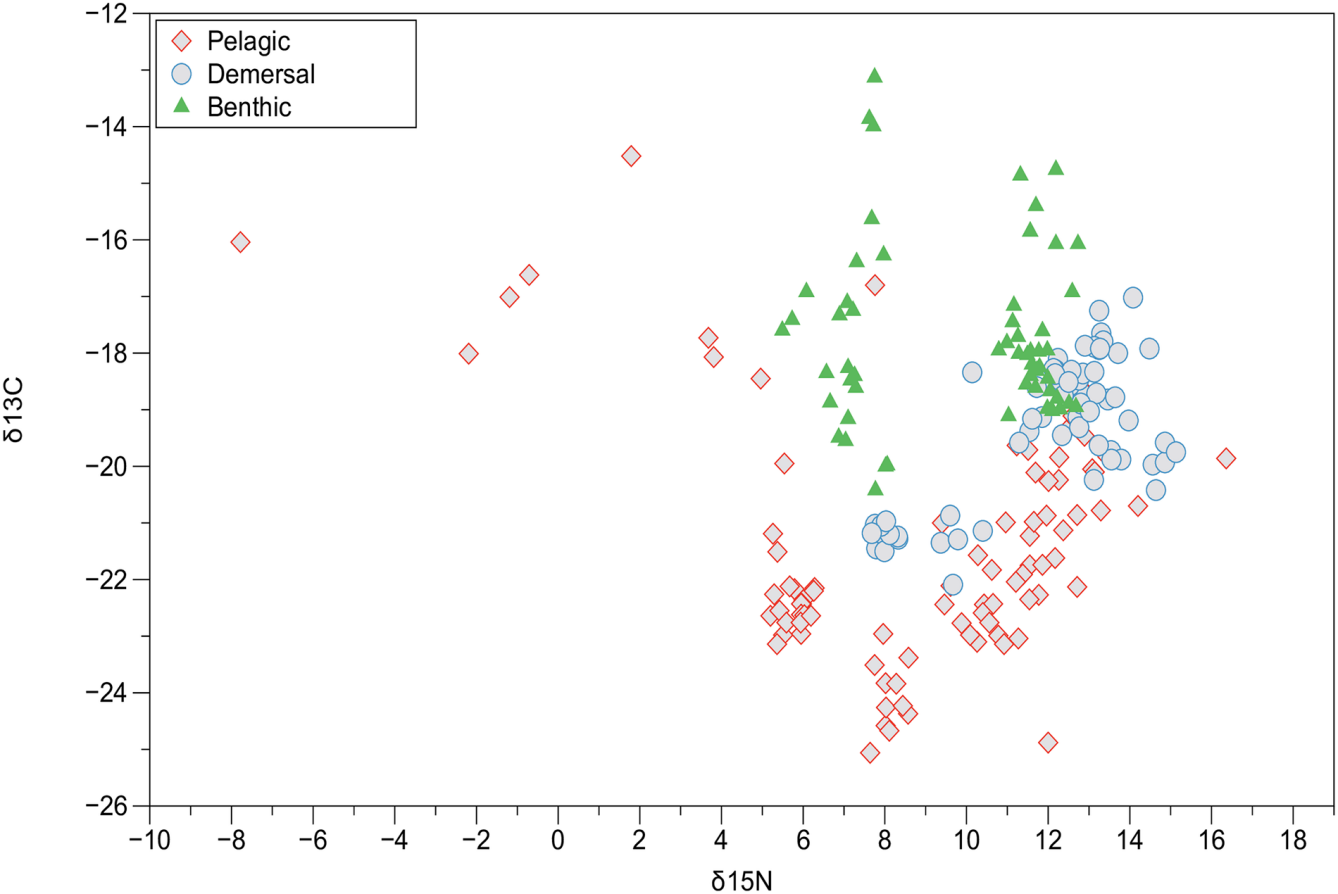
The relationship between stable isotope values of δ^13^ C versus δ^15^ N for organisms from the components of the marine ecosystem at the outer Bay of Fundy, Gulf of Maine.

The scatter plot of δ^13^ C against δ^15^ N is difficult to interpret as indicating a single phytoplankton food source for the different habitats (Fig 3). The upper pelagic trophic levels have lower δ^13^ C values than the planktonic base of their food web, whereas the demersal and benthic species values are more indicative of a planktonic food base.

**Fig 4:**
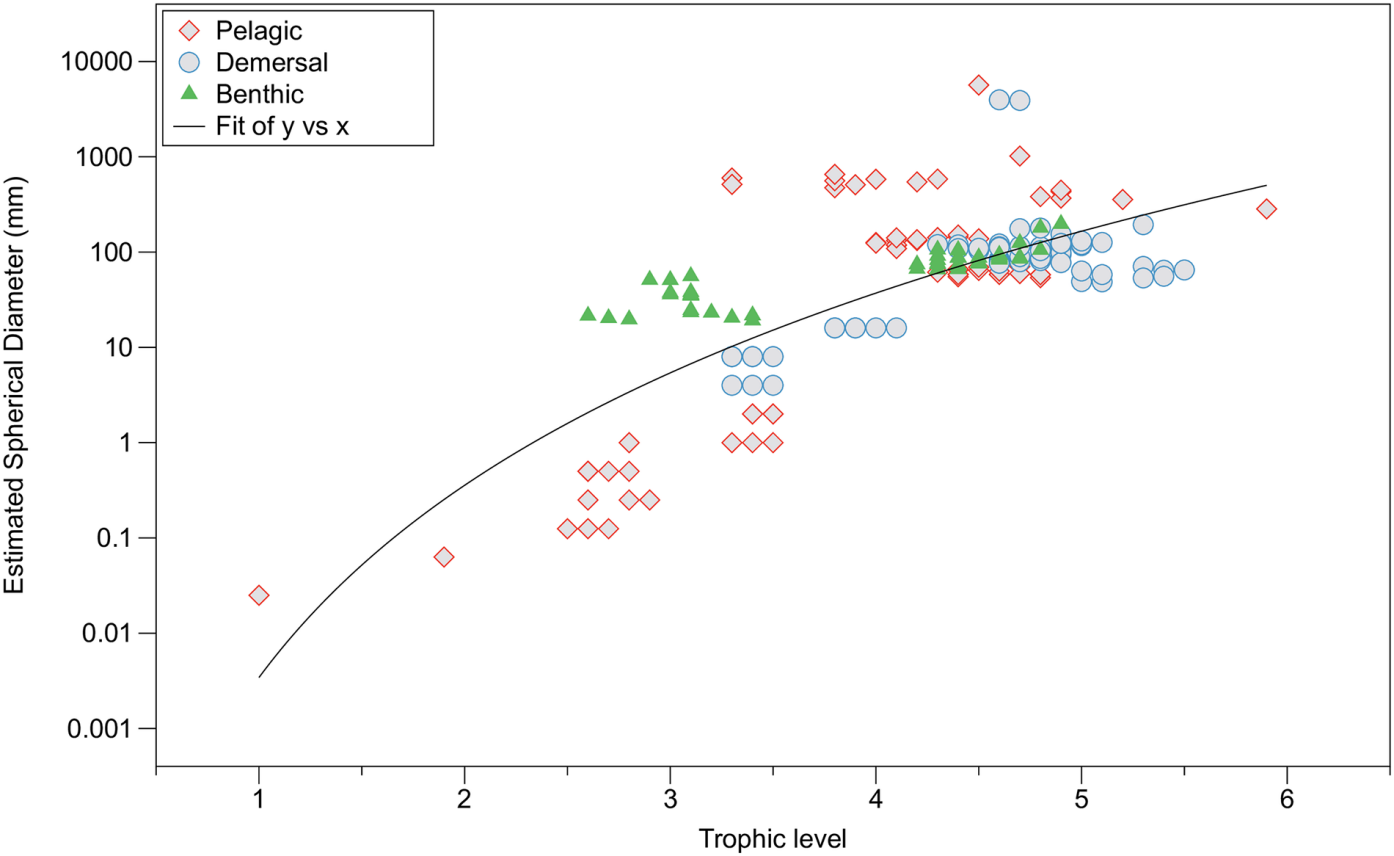
The relationship between the estimated spherical diameter (ESD) of an organism and its calculated trophic level from δ^15^ N values, for that portion of the food web from the outer Bay of Fundy where stable isotopes were measured. Y = 0.003X^6.7^, r^2^ = 0.69, n = 202.

It is important to note that the traditional trophic level concept is an oversimplification because most species have developmental stages that occur at a lower feeding level in the food chain. The unit trophic level (TL1, TL2, etc.) is used here, however, to calculate a measure of biological magnification (BMF) within a food chain. The more realistic approach to trophic phenomenon, as a loose continuum of feeding types, is that used throughout and enables the calculation of the total magnification factor (TMF) and the biomagnification power of the ecosystem. The δ^15^ N isotope technique enables a measure of both a trophic continuum and the more traditional incremental trophic levels [29, 45]. Classifying organisms entirely by size presents another approach for assigning marine organisms to a continuum of feeding types as they develop through various trophic levels. The use of organism size, as a proxy for trophic level, enabled the calculation of a continuous trophic structure comparable to that determined by δ^15^ N (Fig 4).

### Bioconcentration of mercury

It has been known for quite some time that marine life concentrates mercury several orders-of-magnitude above the levels found in “seawater” [89]. The largest increase, in general, occurs between the “dissolved” and particulate fraction, which is distinguished by some arbitrary filter size (usually 0.45μm). This definition of “dissolved”, however, includes smaller planktonic organisms, detritus and resuspended particulates in the filtrate [90]. The difference between the mercury concentration in our smallest “phytoplankton fraction” (25–66μm) sampled and that “dissolved” in seawater, expressed as a bioconcentration ratio or factor (BCF), is three (MeHg) and four (THg) orders-of-magnitude higher than that found in seawater (Table 4). These calculations assume that our 25–66μm size fraction is largely autotrophic and the processes of mercury species ad-, ab- and desorption are at equilibrium. These values fall within the range of oceanic BCF values derived from, albeit, a limited number of field studies that enable this calculation (Table 4; [26,74,91–97]). Earlier experimental work showed that mercury could enter the marine food chain preferentially as MeHg through adsorption to phytoplankton surfaces and absorption into the cytoplasm [68, 69, 98]. To date, experimental studies of mercury uptake from seawater by phytoplankton has been limited by the practical concentrations achievable in the lab. Six categories of marine phytoplankton were subjected to 60 to 90ng MeHg /L concentrations in seawater [99], which is 10^3^ times higher than the values observed here in the Bay of Fundy. This ambiguity in quantifying uptake rates would also apply to flagellates, bacteria, viruses and non-living particles that make up the microbial loop [100–102]. The calculated surface area exposed is also a function of time in mobile organisms and therefore should be a function of their swimming speed [103], which are now readily available for modeling purposes [104, 105].

**Table 4:** Methylmercury (MeHg) and total mercury (THg) bioconcentration factors (BCF) on a wet weight basis between seston/plankton and seawater in the world oceans.

Region	Particles (size)	MeHg	THg	References
N. Gulf of Maine Bay of Fundy, Canada	Plankton (25–66μm net); unfiltered sea water	2.1 X 10^3^	1.3 X 10^4^	This study
Chesapeake Bay, MD., USA	Plankton (153μm net); unfiltered sea water		5.8 X 10^4^	[91].
Guanabara Bay, Brazil	Seston (1.2μm GF/C filter)	2.8 X 10^4^	4.6 X 10^3^	[92].
	Plankton (70–290μm net);	1.2 X10^4^	2.3 X10^3^	[93].
	unfiltered sea water	6.4 X 10^3^	2.2 X 10^3^	
Pacific Ocean off San Diego, CA, USA	Plankton (Bongo; unspecified); unfiltered sea water		3.7 X 10^4^	[94].
Elbe River estuary	Seston (0.45μm GF/C filter); unfiltered sea water		3.1 X 10^4^	[95].
North Sea	Seston (0.2μm nuclepore polycarbonate filter); filtered sea water	1.6 X 10^4^	2.9 X 10^4^	[96].
Belgian Coastal Waters	Seston (0.2μm nuclepore polycarbonate filter); filtered sea water	1.6 X 10^4^	4.6 X 10^4^	[96].
Scheldt estuary, Belgium	Seston (0.2μm nuclepore polycarbonate filter); filtered sea water	5.6 X 10^3^	1.2 X 10^5^	[96].
Mason Bay, S. Korea	Seston (0.4μm GF filter); unfiltered sea water	1.2 X 10^3^	1.1 X 10^3^	[26].
Long Island Sound, NY., USA	Seston (0.2μm filter); filtered sea water	1.6 X 10^4^		[97].
Northeast Atlantic Shelf, USA	Seston (0.2μm filter); filtered sea water	2.0 X 10^4^		[74].

The abundance of particles, both living and inert, increases exponentially as a power function with decreasing size as far as the spectrum has been quantified (>5nm; [102, 106,107]). Particulate surfaces available for metal adsorption in seawater are presumably further extended by association with organic ligands [108], such that >99% of ‘dissolved’ mercury can be complexed by ligands associated with natural ‘dissolved’ organic material [109].

**Fig 5:**
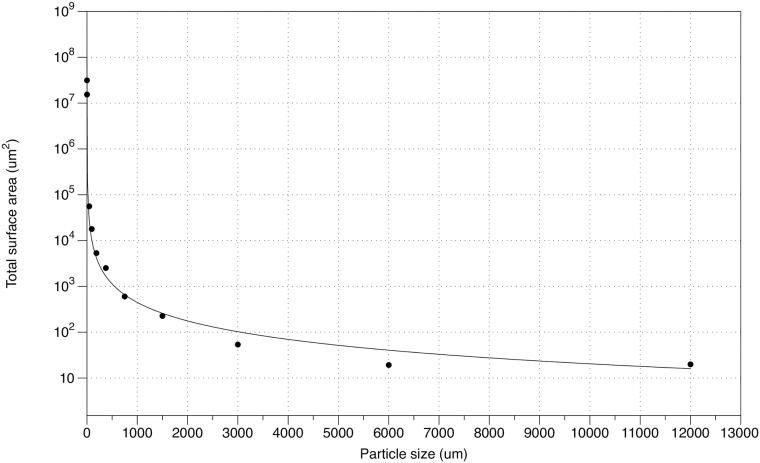
Potential surface area available for mercury sorption over the marine size spectrum calculated from the nekton/plankton categories reported here (16mm—25μm), together with the particulate (1μm ~ 450nm) and colloidal (~ 450nm- 5nm) material from the literature [113, 114]. This particulate fraction includes nanoplankton, bacteria, cyanobacteria, picoeukaryotes, viruses and inert particulates [102].

If the above BCF calculations are based on the abundance of particles per m^3^ of seawater, that is on a volume basis (pg mercury /m^3^ of phytoplankton in seawater)/(pg mercury /m^3^ seawater) rather than on a wet weight basis, the average concentration of MeHg and THg associated with our smallest phytoplankton fraction in the Gulf of Maine is less than that “dissolved” in seawater by 1.7 X 10^−3^ and 6.0 X 10^−3^, respectively. This apparent abrupt change can be explained by taking into account the increase in particle surface area, smaller than 25μm, with decreasing size down to at least 450nm, and probably further down to include the colloids (5nm) and perhaps organic ligands [69,110]. The abundance of particles in the ocean, as measured over a broad size range, is best fitted by a power-law distribution with an exponent of approximately -3, which indicates equal particle volumes between logarithmic size intervals [111,112]. The marine size spectrum calculated from the 10 nekton/plankton categories reported here (16mm—25μm), together with the particulate (1μm ~ 450nm) and colloidal (~ 450nm- 5nm) material derived from the literature [113,114] results in a particle distribution of abundance (No./ml) equal to 1.5*10^−6^ ESD^-3.34^. This is a reasonably close fit to the expected exponent of -3, given the wide size range of the particle and colloid categories. In Fig 5, we illustrate this as a function of particulate surface area (SA) versus size:

$$\text{SA}=1.2*10^{6}{\text{ESD}}^{-1.1},\text{r}=0.98.$$

The presence of this large surface area available in the so-called “dissolved seawater” category, therefore, can explain the perceived abrupt decrease of both MeHg and THg on a seawater volume basis and the increase on an organism wet weight basis (BCF) because of the artificial choice in separating particulates from dissolved concentrations (Table 4).

### Bioaccumulation of mercury

No attempt was made in this study to measure the role played by the autotrophic pico- and nanoplankton of the microbial loop [115,116]. This was due to both problems associated with collecting sufficient material for analysis and the lysing of delicate flagellates in the filtration process. Although Heimburger et al. [117] implicated nano- and picoplankton seasonally in the methylation of mercury in both the euphotic zone and underlying water of the Mediterranean Sea; they were unable to separate them by filtration. Chemical nutrients associated with microbial life are rapidly recycled in the surface waters [118]. It has been estimated that as little as 1–2% of this microbial production reaches the upper trophic levels of fishes [119]. Unfortunately there are no studies on the transfer efficiency of microbial production to the base of the “traditional”phytoplankton-to-fish food chain through protozoans. Nonessential elements, such as mercury that adhere to organic surfaces, are likely to be similarly recycled within the microbial loop.

Biomagnification factors were determined by both the nitrogen isotope and the size fraction methods (Table 5). The size spectrum approach to predicting the prey size works reasonably well at predator sizes smaller than baleen whales. For example, Atlantic white-sided dolphins, harbour porpoise, Atlantic bluefin tuna and swordfish (TL 4–5) are known to feed on herring, mackerel and similar-sized demersal fish [120–123]. Atlantic herring and Atlantic mackerel (TL 4.5), in turn, are known to feed on a size range from large copepods (*Calanus*) (TL 3.1–3.4) to krill-sized (*Meganyctyphanes*) (TL 3.4) prey [124–126]. Krill and pteropods (TL 3.4 to TL3.9) feed on macro- and mesoplankton, such as *Calanus*, *Pseudocalanus* and *Limacina* (TL 2.7–3.1)[127–129], which in turn feed on microplankton [130–133]. The fin, minke and humpback whales sampled are an exception in that they not only feed at the trophic level of dolphins and swordfish sized fish, but also on nekton, the latter dominated by the krill *Meganyctyphanes* [134,135].

**Table 5:** Biomagnification factors (BMF) calculated from both size spectra and nitrogen isotope based trophic levels.

Species/Category	ng MeHg/g wet	ng THg/g wet	%MeHg	ESD (mm)	TL±SD	BMF (size spectrum)	BMF (isotope)
Fin whale	6.61	25.97	25.5	4509	4.6±1.2	0.2–0.3	1.3
Humpback whale	32.24	43.3	74.5	2530	-	0.5–0.6	-
Minke whale	72.54	79.4	91.4	1816	-	1.0–1.4	-
Atlantic White-sided dolphin	510.8	1261.4	40.5	664	-	25–35	-
Harbour porpoise	325.8	606.8	53.7	379	5.1±1.4	16–22	23.2
Common thresher shark	1426.9	1472.4	96.9	1018	4.7	24–33	69.4
Bluefin tuna	495.7	564.9	87.8	860	-	10–14	-
Swordfish	293.9	416.4	70.6	567	3.8±1.4	9–13	34.7
Spiny dogfish	83.9	99.3	84.5	139	4.2±1.2	4–6	6.3
Pollack	15.4	18.7	82.4	81	4.6±1.2	1–1.4	0.93
Atlantic Herring	54.6	59.8	91.3	68	4.5±1.2	3.6–5.0	3.2
Atlantic Mackerel	17.4	21.8	79.8	66	4.6±1.2	1.3–1.8	1.1
Atlantic Cod	27.1	35.2	77.0	135	4.7±1.3	1.5–2.1	1.7
White hake	24.1	29.5	81.7	126	5.0±1.1	1.3–1.8	1.2
Haddock	18.3	32.3	56.7	93	4.8±1.1	1.7–2.3	1.4
Cunner	75.3	79.7	94.5	59	5.2±1.2	5–7	2.9
Yellowtail flounder	23.3	26.9	86.6	100	4.5±1.2	1.3–1.9	1.4
Winter flounder	15.2	21.1	72.0	75	4.4±1.1	1.2–1.7	1.2
American Lobster	27.7	35.9	77.2	86	4.6±1.1	1.9–2.7	1.8
Sea scallops	6.9	26.9	25.7	41	3.1±1.1	2.1–2.9	4.0
Blue mussels	5.3	19.6	27.0	22	3.1±1.3	2.0–2.8	2.9
Nekton 16mm	17.4	26.3	66.2	16	3.9±1.1	3.1–4.4	2.0
Nekton 8mm	5.7	9.0	63.3	8	3.4±1.1	1.5–2.1	1.0
Nekton 4mm	5.1	7.4	68.9	4	3.4±1.1	1.7–2.4	0.84
Macroplankton 2mm	1.9	3.1	61.3	2	3.4±1.1	1.0–1.4	0.36
Macroplankton 1mm	1.0	2.0	50.0	1	3.1±1.3	0.9–1.3	0.31
Mesoplankton 500μm	0.5	1.4	35.7	0.5	2.7±1.1	0.9–1.2	0.32
Mesoplankton 250μm	0.5	1.9	26.3	0.25	2.7±1.1	1.7–2.3	0.38
Microplankton 125μm	0.4	1.8	22.2	0.125	2.6±1.1	2.2	0.48
Microplankton 63μm	0.09	3.4	2.6	0.063	1.0	-	-
Microplankton 25μm	0.05	3.4	1.5	0.025	1.0	-	-

Both the δ^15^ N isotope and size fraction approaches can be used to quantify the continuum of overlapping feeding preferences present in nature. Biomagnification factors (BMF) derived from δ^15^ N values and organism size (ESD) are similar, within the same order-of-magnitude, from ~TL3.4 to the top predators at ~TL5 (Table 5). BMF values, in general, are relatively uniform between 1 and 3 from the 3^rd^ to 4^th^ trophic levels. (Values greater than 1 indicate that biomagnifications has taken place [53]). The initial biomagnification of THg from our “phytoplankton” fraction (25um), using the size-spectra approach, was mainly due to an increase in MeHg concentrations between the meso-to macroplankton categories. Biomagnification of mercury, however, was not detected until the 8mm nekton category using the δ^15^ N isotope approach (Fig 2, Table 5). The overall trends in BMF values, however, calculated with both approaches are in general agreement.

The larger predators, such as Atlantic white-sided dolphins, harbour porpoise, common thresher shark, Atlantic bluefin tuna, swordfish and spiny dogfish, have high BMF values between 4 and 69 by both methods which was not expected from their estimated trophic levels. This leaves their greater age as a possible unaccounted for factor that could modify our estimate of BMF solely based on trophic level.

The baleen whales in general have low BMF values at or below 1. The minke, humpback and fin whales feed predominantly on herring and similar-sized fishes in the water column [122, 136], so their low mercury levels relative to the porpoises, dolphins, swordfish and bluefin tuna may be due to their ability to switch to krill and the larger *Calanus* copepods, depending on prey availability [134] (Table 5). Another possibility is that our surface muscle samples from baleen whales were under representative of deeper muscle mercury levels by being permeated (“marbled”) by lipid reserves. Baleen whales spend part of the year in more tropical waters conserving energy and giving birth but their feeding grounds, and trophic accumulation of mercury, are on the productive northern continental shelves, such as Browns and Georges Bank in the Gulf of Maine [137,138].

**Fig 6:**
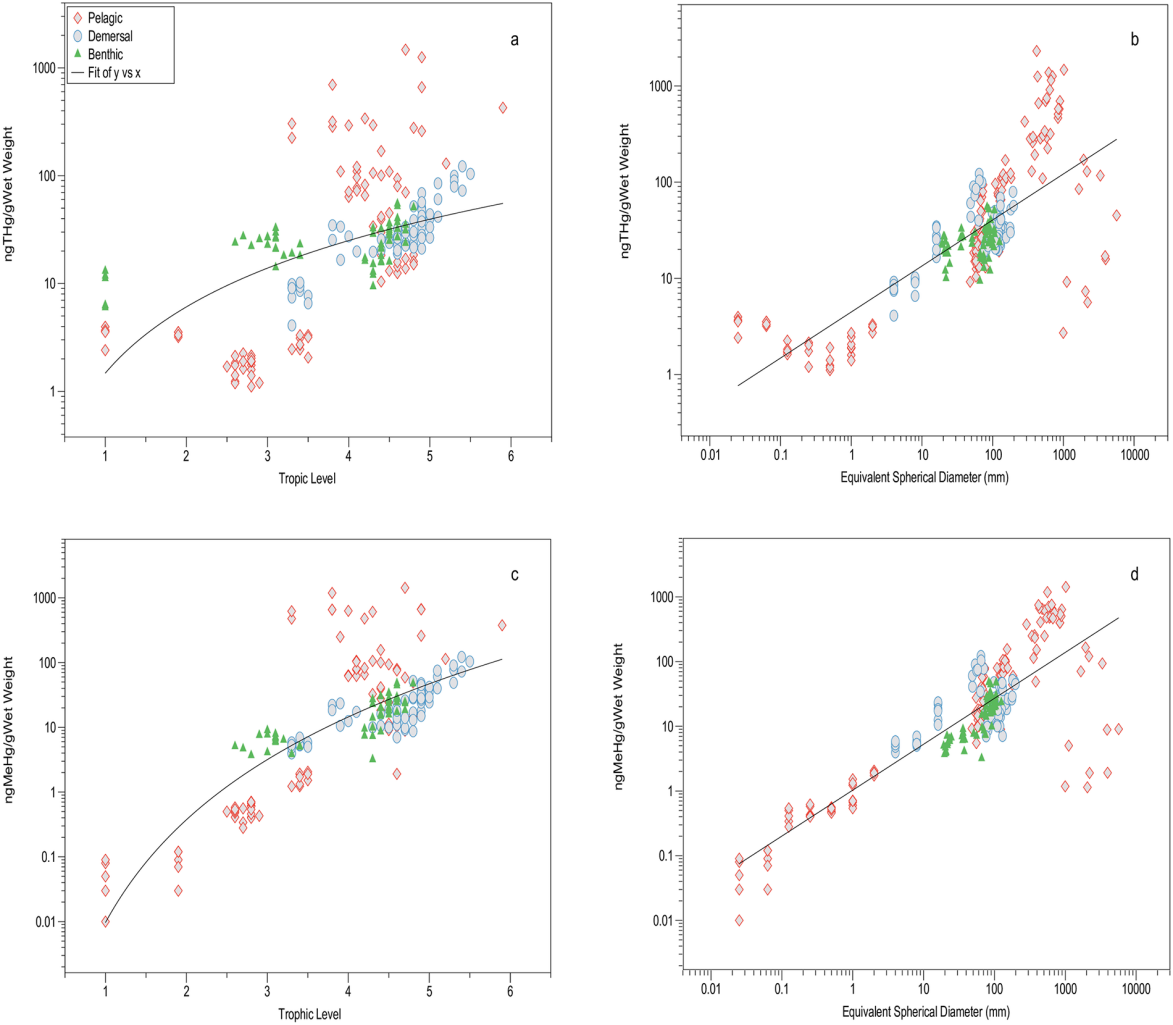
The relationship between ng THg/g wet weight and trophic level (a) and equivalent spherical diameter (b), and between ng MeHg/g wet weight and trophic level (c) and equivalent spherical diameter (d).

Regressions of THg and MeHg concentrations against either trophic level derived from δ^15^ N values or against organism size, were best fitted to power curves with a positive exponent (Fig 6):

$$\left[\text{THg}\right]=0.9{\left(\text{TL}\right)}^{2.5},\;\text{r}=0.49,\;\text{n}=202,\;\text{P}<0.001\left(\text{a}\right),$$

$$\left[\text{THg}\right]=4.3{\left(\text{ESD}\right)}^{0.5},\;\text{r}=0.76,\;\text{n}=270,\;\text{P}<\text{0}.001\left(\text{b}\right),$$

$$\left[\text{MeHg}\right]=0.01{\left(\text{TL}\right)}^{5.3},\;\text{r}=0.79,\;\text{n}=202,\;\text{P}<0.001\left(\text{c}\right),$$

$$\left[\text{MeHg}\right]=1.03{\left(\text{ESD}\right)}^{0.71},\;\text{r}=0.84,\;\text{n}=270,\;\text{P}<0.001\left(\text{d}\right).$$

There are two main features that stand out from the calculated BMF values; first, biomagnification of mercury does not appear to commence noticeably in the Gulf of Maine, despite the rise in MeHg levels, until the 3rd trophic level, somewhere after the mesoplankton, and, secondly, biomagnification values generally remains low until the top trophic levels, occupied by the large pelagic fish, porpoise and dolphin categories (Table 5). Our observations on the lower trophic levels (Fig 2) are consistent with previous studies showing that methylated mercury is the predominant form bioaccumulated up the trophic chain both from experimental [15,16,139] and field studies [21,22,140]. This initial bioaccumulation of the methylated form, relative to THg, starting from the “phytoplankton” through mesoplankton to krill level, is difficult to explain from an entirely food chain perspective given the extremely low methylmercury levels in both “seawater” and their food source [9,11,141]. Furthermore, the time available for uptake from “seawater” is brief with the generation times at this latitude ranging from12 hours for diatoms [142], ~20 days for mesoplankton [143,144] and ~6 months for macroplankton [145].

In the Gulf of Maine planktonic/nektonic food chain THg concentrations are relatively stable, 2.4 to 3.6ng/g wet weight, from the phytoplankton-dominated microplankton (25μm) through to the macrozooplankton (2.0mm), such as *Calanus hyperboreus* (Table 3), which explains our low biomagnification factors (BMF, Table 5). The MeHg levels increase incrementally from 0.12ng/g wet weight in the microplankton to 14.5ng/ g wet weight in the largest nektonic category (16.0mm). However, the inorganic component of THg content decreases from 90–96% in the microplankton to 76–80% in the mesozooplankton to 36–56% in the macrozooplankton and 32–46% in the nektonic categories.

There are, therefore, several interpretations of these observations with MeHg being either preferentially taken up directly from “seawater” or that inorganic mercury is methylated within the organisms or both. It is time to consider the internal conversion of mercury by zooplankters more seriously given the low levels of MeHg measured in seawater in this study (58.4±22.5pg/L), and the known strong bonding of mercury to surfaces, together with the enormous competing surfaces available for its adsorption in seawater [14,109] (Fig 5).

The answer may be that MeHg accumulations are not totally due to feeding at the lower levels of the food chain. Pucko et al. [146] postulated an enhanced production of MeHg from inorganic mercury within the copepod *Calanus hyperboreus* based on similar observations of low values of MeHg in both seawater and its filtrate (0.7 μm); the latter being the assumed food source of these copepods. The likely initial source would be MeHg production by gut microbes in the zooplankton from inorganic mercury. This source of MeHg production would then bioaccumulate progressively up the food web. A number of studies have shown that methylation of mercury occurs in the guts of terrestrial insects [147], earthworms [148] and fresh water fish [149]. Sulphate- and iron- reducing bacteria are usually implicated in mercury methylation, however, recent work with specific genetic markers has diversified the capable microbes to include methanogens and a wide variety of *Firmicutes* [150]. It has been found in freshwater studies that a bacterial diet, determined by fatty acid composition, is a better predictor of MeHg accumulation in zooplankton although bacteria are not as nutritional as an algal diet [151]. It is also possible that other bacteria are capable of methylating inorganic mercury. Studies on mercury methylation within zooplankter guts are needed to investigate this possibility.

### Biomagnification power

Borga et al. [53] explored many of the pitfalls involved in comparing biomagnification powers or total magnification factors (TMF) across ecosystems; such as 1) tissues measured being representative of the entire organism, 2) number of trophic levels measured, 3) representative sampling of trophic levels, 4) sufficient overall sample size of > 60 measurements to mention the main concerns. The results of the present study, together with selected marine studies representing reasonable sample sizes and number of trophic levels, are listed in Table 6.

**Table 6:** Bioaccumulation power (b) of mercury (THg or MeHg) in comprehensive marine food web studies, where Log10[mercury] = b(δ15N) − a and the total magnification factor is (TMF) = 10^b^.

Ecozone	Mercury	b	a	r^2^	p	n	TMF*	Categories**	References
Arctic (Lancaster Sound)	THg	0.20	-3.3	?	<0.01	112	1.6	IA, 3Z, 9BI, 2F, 8B, 5M	[21]
Arctic (N. Baffin Bay)	THg	0.20	-3.4	0.68	≤0.001	92	1.6	IA, 2Z, 1BI, 1F, 8B, 1M	[22]
	MeHg	0.22	-3.9	0.74	≤0.001	76	1.7		
Arctic (SE Beaufort Sea)	THg	0.26	-4.0	0.9	<0.01	192	1.8	2Z, 6F, 1M	[23]
	MeHg	0.30	-4.5	0.8	<0.01	37	2.0		
Subarctic (Baltic Sea)	THg	0.18	-3.0	0.57	≤0.001	25	1.5	P, 1Z, 1BI, 1F	[24]
Temperate (Masan Bay, Korea)	THg	0.12	+0.04	0.37	<0.05	85	1.3	8BI, 12F	[26]
	MeHg	0.17	-0.91	0.46	<0.05		1.5		
Temperate (Gulf of St.Lawrence)	THg	0.17	-0.29	0.5	≤0.001	285	1.5	4Z, 10BI	[25]
	MeHg	0.24	-1.53	0.51	≤0.001		1.7		
Temperate (Outer Bay of Fundy)	THg	0.11	+0.16	0.39	≤0.001	202	1.3	2P, 5Z, 3N, 11F, 2S, 5M	This study
	MeHg	0.20	-1.03	0.62	≤0.001		1.6		
Subtropical (Gulf of Farallones, CA, USA)	THg	0.32	?	?	≤0.001	56	2.1	Z, 2F, 4B, 1M	[27]
Tropical (Guanabara Bay)	THg	0.19	-0.52	0.36	0.0001	86	1.5	SE, 2BI, 16F	[28]

*Most studies have used the slope b of the Log_10_[mercury] versus δ^15^ N equation, so the base 10 is used to calculate the TMFs [53].

** Categories are P for phytoplankton, SE for seston, IA for ice algae, Z for zooplankton, N for nekton, BI for benthic invertebrates, F for fish, S for sharks, B for birds and M for mammals.

The biomagnification power, the slope b of the equation Log10 [Hg] = b(δ15N) − a, of the Bay of Fundy food web values of 0.11 and 0.20 for THg and MeHg concentrations, respectively, fall in the narrow ranges of previous studies (Table 6). This endorses the view that Campbell et al. [22] put forward, and the recent review of Chetelat et al. [152], that the factors involved in marine food chain biomagnification of THg, and MeHg in particular, are similar over a wide range of environments from polar to temperate to tropical latitudes. Lavoie et al. [153] recently collated studies, of various trophic extent, on food chain mercury bioaccumulation in both freshwater and marine environments and concluded that there was a decrease in the slope b from 80°N to ~45°S. The basis for this generalization, however, appears to be due to low values obtained from lake studies in the southern hemisphere.

The benthic, demersal and pelagic food webs of a coastal marine ecosystem, such as in the northern Gulf of Maine, are all heavily reliant on phytoplankton production at their base. In fact, most marine organisms have planktonic larval stages that directly take advantage of the primary production in the euphotic zone. This diversification into bottom, near-bottom and pelagic life styles leads to trophic diversification of the shelf ecosystem but does not appear to alter the relationship between mercury accumulation and trophic level (Fig 6). Marine studies published thus far do not support a latitudinal or inshore-offshore change in biomagnification power, although more detailed studies are needed, particularly in the tropical and subtropical latitudes.

## Conclusions

Fine-scale, size sampling of a marine temperate, food web demonstrates that 98% of the mercury is inorganic at the phytoplankton level but this proportion is reduced to less than 50% at the macrozooplankton or third trophic level. Food-chain biomagnification of methylmercury dominates, thereafter, with BMFs >1 to the uppermost trophic levels, such as Atlantic bluefin tuna, Atlantic white-sided dolphins and common thresher sharks. The apparent abrupt increase in mercury concentration between “seawater” and plankton (bioconcentration) can be explained as a gradual physical/chemical phenomenon by accounting for all particulate surfaces in seawater. Our δ^13^ C isotope values were found inadequate to distinguish between pelagic, demersal and benthic components of the Bay of Fundy food web, however, collated published production studies indicate that water-column production overwhelmingly dominates as a carbon source for the area. Pelagic, demersal and benthic components are reliant on the same water-column production. δ^15^ N isotope and organism size are both valid continuous-scale measures of trophic level, with the possible exception of baleen whales, which feed over a broader range of prey sizes. The biomagnification power of mercury in the northern Gulf of Maine food web is similar to that measured in tropical, subtropical, other temperate and arctic marine ecozones suggesting that common physical/chemical and trophic properties determine bioaccumulation.

## Supporting information

S1 Text Tissue levels of larger fish and marine mammals measured to estimate their body mercury concentrations. (DOCX)

S2 Text Discussion of tissue ratios of carbon isotopes, δ^13^ C, in the Bay of Fundy food chain. (DOCX)

S1 Table Depth distribution of methylmercury and total mercury concentrations in unfiltered seawater at stations located across the entrance to the Bay of Fundy, Gulf of Maine. (XLSX)

S2 Table Methylmercury and total mercury levels measured in planktonic to nektonic organisms collected at the mouth of the Bay of Fundy, Gulf of Maine, in August of 2000 and June of 2001. (XLSX)

S3 Table Methylmercury and total mercury levels, together with δ^13^ C and δ^15^ N values, measured in planktonic to nektonic organisms collected at the mouth of the Bay of Fundy, Gulf of Maine, in August of 2002. (XLSX)

S4 Table Methylmercury, total mercury, δ^13^ C, δ^15^ N, ESD, trophic level and other variables for fish and shellfish species collected for the Bay of Fundy study, Gulf of Maine. (XLSX)

S5 Table Methylmercury and total mercury levels measured in marine mammals collected in the Gulf of Maine and environs between 1999–2004. (XLSB)
