# Supplementary material for: Bioaccumulation of methylmercury within the marine food web of the outer Bay of Fundy, Gulf of Maine
Source: PLoS One. 2018 Jul 16;13(7):e0197220. doi: 10.1371/journal.pone.0197220 (PMC6047777; doi:10.1371/journal.pone.0197220)
Supplement: S1 Text — (DOCX) [file pone.0197220.s001.docx]

S1 Text. Tissue levels of the larger fish and marine mammals measured to estimate their body mercury concentrations.

The total mercury concentration in the muscle tissue of our swordfish >100kg and <100kg was 92(56-1196) and 73(36-141) ng/g wet weight, respectively. These values fall within the range reported for swordfish caught in the N. Atlantic between 2004 and 2005 [1] and earlier in the 1970s off Nova Scotia [2]. Mercury content of bluefin tuna muscle reported here is 768 (632-914)ng/g wet weight for fish between 313 to 379kg. These values are higher than those obtained from fish of undetermined size caught in summers off New Jersey, USA, between 2005 and 2008 [3] but similar to values for fish caught off Nova Scotia in 1970 [4].

The present median and range values for harbour porpoise of 324 (46-972)ng MeHg/g wet and 430 (164-916)ng THg/g wet in muscle, 341 (186-1540)ng MeHg/g wet and 1165 (377-45900)ng THg/g wet in liver and 19 (2-69)ng MeHg/g wet and 47 (9-900)ng THg/g wet in blubber are within the range of values previously reported for the Gulf of Maine and approaches in the 1970s [5]. The mercury levels in white-sided dolphins are similar to levels in the harbour porpoise (Table 3). The mercury levels in baleen whales were lower than found in porpoises and dolphin, which may reflect their ability to feed over a wider range of prey sizes from nekton to herring-sized fish [6,7]. Another explanation is that the muscle tissues of baleen whales analyzed here may have been too superficial and included marbled fatty tissue.

References:

1. Branco V, Vale C, Canario J, Neves dos Santos M (2007) Mercury and selenium in blue shark (*Prionace glauca*, L. 1758) and swordfish (*Xiphias gladius*, L. 1758) from two areas of the Atlantic Ocean. Environmental Pollution 150: 373-380.

2. Beckett JS, Freeman HC (1974) Mercury in swordfish and other pelagic species from the western Atlantic Ocean. NOAA Technical Report NMFS SSRF-675: 154-159.

3. Burger J, Gochfeld M (2011) Mercury and selenium levels in 19 species of saltwater fish from New Jersey as a function of species, size and season. Science of the Total Environment 409: 1418-1429.

4. Bligh EG, Armstrong FAJ (1971) Marine mercury pollution in Canada. International Council Exploration of the Sea. C.M. 1971/E 34, 13p.

5. Gaskin DE, Stonefield KI, Suda P, Frank R (1979) Changes in mercury levels in harbor porpoises from the Bay of Fundy, Canada, and adjacent waters during 1969-1977. Archives Environmental Contamination and Toxicology 8: 733-762.

6. Mitchell E (1974) Trophic relationships and competition for food in northwest Atlantic whales. Proceedings Canadian Society of Zoologists Annual Meeting., June 2-5, pp 123-132.

7. Harding G (2013) Toxic Chemical Contaminants: Review. State of the Gulf of Maine Report. Companion document to Toxic Chemical Contaminant theme paper. 59pp. [www.gulfofmaine.org/stateofthegulf](http://www.gulfofmaine.org/stateofthegulf).
