# Supplementary material for: Bioaccumulation of methylmercury within the marine food web of the outer Bay of Fundy, Gulf of Maine
Source: PLoS One. 2018 Jul 16;13(7):e0197220. doi: 10.1371/journal.pone.0197220 (PMC6047777; doi:10.1371/journal.pone.0197220)
Supplement: S2 Text — (DOCX) [file pone.0197220.s002.docx]

S2 Text. Tissue ratios of carbon isotopes, δ^13^ C, in the Bay of Fundy food chain.

The 25 to 63µm microplankton category, consisting mainly of diatoms and flagellates, had δ^13^ C values of -16.2±1.2 ‰ that indistinguishable from those obtained from the attached rockweed *Ascophyllum* (-16.1±0.4‰). The 63 to 125µm size category, mainly diatoms, had δ^13^ C values lower than the preceding size category at -18.4±0.9‰. The largest microplankton category (125 to 250µm), which is a 50% mix of diatoms and crustaceans, has δ^13^ C values lower than the preceding category at -22.3±0.2‰. The latter two microplankton categories have δ^13^ C values within the range of many previous studies in NW Atlantic coastal areas [1-5] but higher than those from the neighbouring Gulf of St. Lawrence and Scotian Shelf [6-8] The meso-, macro-plankton and nekton categories had δ^13^ C values lower at -22.2±0.4‰, -23.4±1.0‰ and -21.1±0.8‰, respectively, than expected from a combined microplankton food source of -17.3±1.5‰. This suggests that there may be an unmeasured food source at the base of the planktonic food chain deficient in heavy carbon.

Sources of carbon for the planktonic/nektonic food web of the Gulf of Maine other than phytoplankton are the degradation products of macrophytes, eelgrass, low and high salt-marsh plants, benthic algae and detrital or bottom sediments. The bladder wracks (*Fucus* *vesiculosus* ) measured in our study also had a significantly higher δ^13^ C value of -13.7±0.5, that was very different from values for other trophic levels documented here of either benthic, demersal or pelagic food paths. Benthic diatoms, not measured here, have δ^13^ C values of between -18.5 and 13.2‰ in the Bay of Fundy [1] that overlap with our two smaller-sized microplankton categories and other studies [9-10]. Water column and sediment bacteria should be ^13^ C enriched relative to their food source [11], which is predominantly phytoplankton in the Bay of Fundy [12]. Eelgrass (*Zostera marina*) also has high δ^13^ C values ranging from -14 to -10‰ [4,13]. Schwinghamer et al. [1] reported values of -13.1‰ for the lower salt-marsh grass, *Spartina* *alterniflora*, in the Bay of Fundy. High salt-marsh plants, such as sedges, have very low δ^13^ C values of -28.0±1.3‰ [14,15]. The majority of this macrophyte biomass is rafted-up over the high tide mark by winter storms and either accumulates in tidal salt marshes [16] or decomposes gradually as the winter weather ameliorates with some organics leaching back into the near-shore waters [17]. However, Cranford et al. [18] found that particulates derived from salt marsh grass decomposition are retained in the upper reaches of the Bay of Fundy by the action of the local hydrography. This finding substantiates the results of sedimentation studies where the upper reaches of the Bay of Fundy were found to be the major repository for erosive particulates [19].

Another explanation for our anomalous δ^13^ C values in the microplankton categories could be that, with their shorter turnover time, the organisms we collected were not representative of the time interval over which the mesoplankton and nekton developed. The seasonal ^13^ C content of phytoplankton is known to change with highest values occurring during the spring or late-summer blooms [5, 20, 21]. Diatoms blooms in the spring and during the well-mixed summer waters over Georges Bank are enriched in ^13^ C at -19 to -15‰ δ^13^ C compared to -25 to -21‰ over the rest of the year [22]. The samples used here for isotope analysis were collected in late summer, which may account for the high δ^13^ C values in the 25 to 63µm microplankton fraction. There is not enough information available in the present study, therefore, for the δ^13^ C values to be used more definitively as an indication of primary dietary source.

Herring (-21±0.8‰) and mackerel (-19.8±0.9‰), predominantly pelagic feeders [23, ], have δ^13^ C values to be expected from a zooplankton/nekton food source. The demersal fish, benthic shellfish and flatfish δ^13^ C values measured here also are consistent with a planktonic food source although it is possible that there is an indirect augmentation of a phytoplankton based food-chain by detritus consumption by the larger planktonic and nektonic size categories.

References:

1. Schwinghamer P, Tan FC, Gordon Jr. DC (1983) Stable carbon isotope studies on the Pecks Cove mudflat ecosystem in the Cumberland Basin, Bay of Fundy. Canadian Journal of Fisheries and Aquatic Science 40(Suppl. 1): 262-272.

2. Gearing JN, Gearing PJ, Rudnick DT, Requejo AG, Hutchins MJ (1984) Isotopic variability of organic carbon in a phytoplankton-based, temperate estuary. Geochimica et Cosmochimica Acta 48: 1089-1098.

3. Fry B (1988) Food web structure on Georges Bank from stable C, N, and S isotopic compositions. Limnology & Oceanography 33: 1182-1190.

4. Stephenson RL, Tan FC, Mann KH (1986) Use of stable carbon isotope ratios to compare plant material and potential consumers in a seagrass bed and a kelp bed in Nova Scotia, Canada. Marine Ecology Progress Series 30: 1-7.

5. Wainright SC, Fry B (1994) Seasonal variation of the stable isotope compositions of coastal marine plankton from Woods Hole, Massachusetts, USA, and Georges Bank. Estuaries 17: 552-560.

6. Tan FC, Strain PM (1979) Carbon isotope ratios of particulate organic matter in the Gulf of St. Lawrence. Journal of the Fisheries Research Board Canada 36: 678-682.

7. Tan FC, Strain PM (1983) Sources, sinks and distribution of organic carbon in the St. Lawrence Estuary, Canada. Geochimica et Cosmochimica Acta 47: 125-132.

8. Mills EL, Pittman K, Tan FC (1984) Food-web structure on the Scotian Shelf, eastern Canada: a study using ^13^ C as a food chain tracer. Rapports et Proces-verbaux des Reunions Conseil Internationel Exploration de la Mer 183: 111-118.

9. Haines EB (1976) Stable carbon isotope ratios in the biota, soils and tidal water of a Georgia salt marsh. Estuarine Coastal Marine Science 4: 609-616.

10. Riera P, Richard P (1996) Isotope determination of food sources of *Crassostrea gigas* along a trophic gradient in the estuarine bay of Marennes-Oleron. Estuarine Coastal Shelf Science 42: 347-360.

11. Hullar MAJ, Fry B, Peterson BJ, Wright RT (1996) Microbial utilization of estuarine dissolved organic carbon: a stable isotope tracer approach tested by mass balance. Applied Environmental Microbiology 62: 2489-2493.

12. Prouse N.J, Gordon Jr. DC, Hargrave BT, Bird CJ, McLachlan J, et al. (1984) Update on the marine environmental consequences of tidal power development in the upper reaches of the Bay of Fundy. Canadian Technical Report of Fisheries Aquatic Science No. 1256: 65-95.

13. Kang CK, Sauriau P-G, Richard P, Blanchard GF (1999) Food sources of the infaunal suspension-feeding bivalve *Cerastoderma edule* in a muddy sandflat of Marennes-Oleron Bay, as determined by analyses of carbon and nitrogen stable isotopes. Marine Ecology Progress Series 187: 147-158.

14. Peterson BJ, Howarth RW, Garritt RH (1985) Multiple stable isotopes used to trace the flow of organic matter in estuarine food webs. Science 227: 1361-1363.

15. Wainright SC, Weinstein MP, Able KW, Currin CA (2000) Relative importance of benthic microalgae, phytoplankton and the detritus of smooth cordgrass *Spartina alterniflora* and the common reed *Phragmites australis* to brackish- marsh food webs. Marine Ecology Progress Series 200: 77-91.
